# PD-L1 expression in squamous cervical carcinomas of Mozambican women living with or without HIV

**DOI:** 10.1038/s41598-024-63595-7

**Published:** 2024-06-05

**Authors:** Lucília Lovane, Satish Tulsidás, Carla Carrilho, Christina Karlsson

**Affiliations:** 1 Pathology Department, Maputo Central Hospital, Maputo, Mozambique; 2 School of Medical Sciences, Faculty of Medicine and Health, Örebro University, Örebro, Sweden; 3 Faculty of Medicine, Eduardo Mondlane University, Maputo, Mozambique; 4 Medical Oncology Service, Maputo Central Hospital, Maputo, Mozambique; 5 School of Health Sciences, Faculty of Medicine and Health, Örebro University, Örebro, Sweden

**Keywords:** Cancer, Biomarkers, Oncology

## Abstract

Programmed death-ligand 1 (PD-L1) is overexpressed in squamous cervical cancer (SCC) and can be used for targeted immunotherapy. The highest mortality rates of SCC are reported in sub-Saharan Africa, where Human immunodeficiency virus (HIV) prevalence is high. In Mozambique most SCC patients present at advanced stages. Thus, there is a need to introduce new treatment options. However, immunocompromised patients were frequently excluded in previous clinical trials. Our aim was to determine if PD-L1 expression in SCC is as prevalent among women living with HIV (WLWH) as among other patients. 575 SCC from Maputo Central Hospital were included. HIV status was available in 266 (46%) cases PD-L1 expression was scored through tumour proportion score (TPS) and combined positive score (CPS). PD-L1 was positive in 20.1% of the cases (n = 110), TPS (score ≥ 25%) and in 26.3% (n = 144), CPS (score ≥ 1). Stratifying according to the HIV status, WLWH were TPS positive in 16.7%, compared to 20.9%, p = 0.43, and concerning CPS 21.1% versus 28.7%, p = 0.19, respectively. PD-L1 status was not influenced by stage, Ki-67 or p16, CD8 expression influenced only CPS status. Our data indicates that the documented effect of PD-L1 therapy on SCC should be confirmed in randomized clinical trials in an HIV endemic milieu.

## Introduction

It is well established that persistent infection with a high-risk strain of human papillomavirus (hr-HPV) is the primary etiological agent for cervical cancer (CC)^1^. Squamous cell carcinoma (SCC) is the most frequent histological type. The hr-HPV strains 16 and 18 are the most common in cancer tissue^2^. Even though CC is a preventable disease, it remains the fourth leading cancer cause and fourth most common cancer cause-related death among women worldwide^3^. Estimated new cases and deaths in 2020 were 604,000 and 341,000 respectively^4^. The highest incidence and mortality rates are reported in low- and middle-income countries (LMIC), including the sub-Saharan region. Within the sub-Saharan African region, in the eastern Africa this tumour is the first leading cause of cancer^3–6^, due to lack of effective screening programs, qualified human resources and health facilities, low literacy, low socioeconomic status, and extreme poverty^7,8^, along with high prevalences of HPV and HIV^2,9^. Mozambique is one of the eastern-southern African countries with high prevalence of HPV^10–12^ and HIV^13–15^.

Furthermore, in these settings, this cancer is diagnosed in advanced stage^16–19^, most of them with no option for curative therapy^20^, and managed with palliative care based on chemotherapy and/or radiotherapy, to which many of the patients are resistant or have recurrence^21,22^. The lack of adequate treatment and/or delays in treatment after the diagnosis is established contributes to a low overall survival, i.e. 44.5% in 3 years^8^. In respect to all mentioned factors, the immunotherapy could be used for better outcomes in these settings.

The discovery of the immune checkpoints and their inhibitors programmed death 1 (PD-1), programmed death ligand 1 (PD-L1) and cytotoxic T-lymphocyte–associated protein 4 (CTLA-4), has improved the cancer treatment^23^. The immunotherapy based in immune checkpoints inhibitors (ICI) is growing, and PD1/PD-L1 inhibitors are used to treat many cancer types^21,24,25^. Pembrolizumab has shown to be effective in an advanced, metastatic, and recurrent CC expressing PD-L1, as well as nivolumab^22,26–30^. However, in the clinical trials immunocompromised patients were in the past excluded^31^. In some studies where people living with HIV were included, they have shown safety using the ICI immunotherapy^32–36^ with limited and minimal side effects. Thus, immunotherapy using ICI could be one good alternative therapy of advanced cervical cancer in HIV endemic settings.

For ICI immunotherapy with some specific drugs as Pembrolizumab and Cemiplimab, the diagnostic companion of PD-L1 positive immunohistochemistry is a must, while for other drugs the PD-L1 assay is optional^21,37–39^, although it is classified as an imperfect biomarker for response prediction^40^, as shown that some patients with high expression of PD-L1 had no response to the ICI´s therapy and some cancers with low expression of PD-L1 had a long lasting response^40,41^.

The PD-L1 scoring cut-offs are not uniform for all types of available clones, leading to variability in the interpretation^21,42,43^, interobserver variability and reliability within the same clone^44^. Furthermore, there are controversy regarding the interchangeability of PD-L1 clones type approved as companion for some specific drugs in specific types of cancer, although some studies demonstrated a strong correlation of immunohistochemical expression between some clones such as 22C3 and SP263^45,46^.

In this study we aimed to determine PD-L1 expression in cervical SCC of Mozambican patients with specific emphasis on the influence of HIV status on PD-L1 expression. This would set the fundamentals for a possibility to perform a randomized clinical trial concerning the efficacy of PD-L1 therapy in women living with HIV (WLWH) with advanced CC.

## Results

Patients’ characteristics are described in Table 1. The mean age was 49.9 ± 11.8 years [13–95 years) and the median age of 49.5 years. Information of HIV status was available in 266/575 (46.3%) of invasive SCC, and from that, 147/266 (55.3%) cases were non-HIV women and 119/266 (44,7%) were WLWH.

**Table 1:** Overall patient clinical data.

Patient characteristics		
Age (years)		
Average ± standard deviation	49.9 ± 11.8	
Median	49.5	

	No	%
FIGO stage		
Early stage (1B1-IIA)	30	5.2
Advanced stage (IIB-IVB)	213	37.0
No information	332	57.8
Total	575	100
HIV status		
HIV negative	147	25.6
HIV positive	119	20.7
No information of HIV status	309	53.7
Total	575	100
PD-L1 expression		
TPS		
< 25% tumour cells	437	79.9
≥ 25% tumour cells	110	20.1
Total	547	100
CPS		
< 1	403	73.7
≥ 1	144	26.3
Total	547	100
p16 expression		
Negative (score 0)	16	2.8
Positive, partial stain (score1)	5	0.9
Positive, complete (score 2–3)	546	96.3
Total	567	100
CD8		
Score 0	2	0.41
Score 1–2	63	11.2
Score 3–4	498	88.4
Total	563	100

### PD-L1

**Figure 1:**
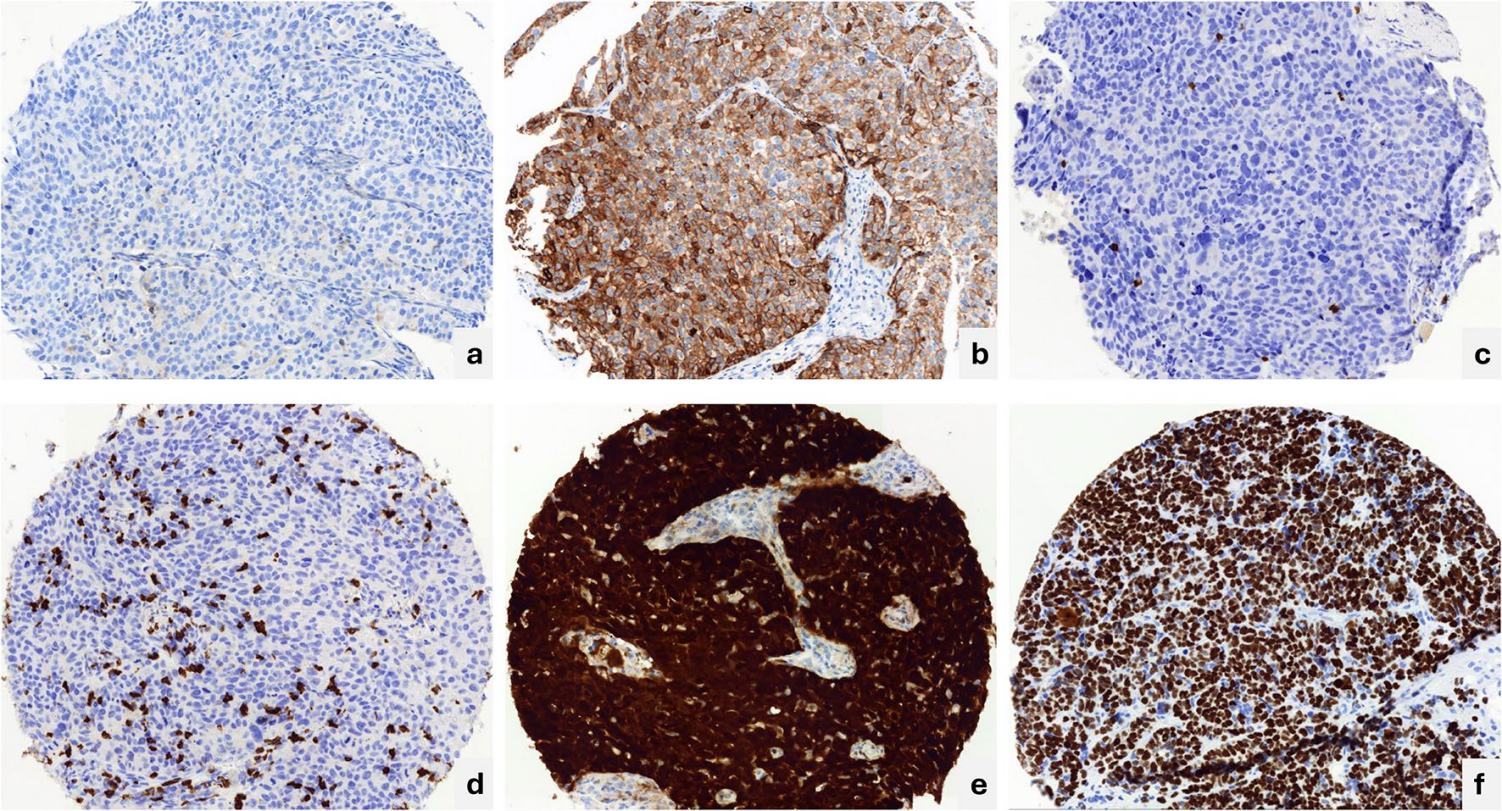
Immunohistochemistry of biomarkers included in the study. PD-L1 (SP263) expression: (**a**) TPS < 25% and CPS < 1% negative; (**b**) TPS ≥ 25% and CPS ≥ 1 positive. CD8 (C8/144B) expression (**c**). positive, score 2; (**d**) positive, score3; (**e**) p16 (E6H4) positive, score 3; (**f**) Ki-67 (MIB-1), score 3. All micrographs taken at 40X.

Twenty-eight (28) cases were excluded from the PD-L1 analysis due to the loss of tissue cores in the TMA construction and/or immunohistochemistry procedures, which resulted in 547 cases evaluable for PD-L1. The PD-L1 expression was positive in 110/547 (20.1%) of the cases in tumour proportion score (TPS) cut-off ≥ 25% and in 144/547 (26.3%) when evaluated in combined positive score (CPS) ≥ 1 (Fig. 1).

When stratified according to the HIV status, 143/147 (97.3%) and 114/119 (95.8%) cases had evaluable TMA cores available in the non-HIV women and in the WLWH groups, respectively. The PD-L1 expression according to the TPS was positive in 30/143 (20.9%) and 19/114 (16.7%) p = 0.4264, in non-HIV women and WLWH groups respectively. The PD-L1 was positive in the CPS in 41/143 (28.7%) and in 24/114 (21%) p = 0.1940 in non-HIV women and WLWH groups respectively. No statistically significant differences were observed in PD-L1 expression in both non-HIV women and WLWH groups (Table 2).

**Table 2:** PD-L1 expression according to HIV status.

	Non-HIV		Women living with HIV		
Age (median, years)					
Average age	53.7 ± 10.8		45.9 ± 10.8		
Median	54		46.5		

	No	%	No	%	
FIGO stage					
Early stage (1B1-IIA)	19	13.0	6	5.0	
Advanced stage (IIB-IVB)	105	71.4	102	85.7	
No information	23	15.6	11	9.3	
Total	147	100	119	100	
PD-L1 expression					p value
TPS					
< 25% tumour cells	113	79	95	83.3	0.4264
≥ 25% tumour cells	30	21	19	16.7	
Total	143	100	114	100	
CPS					
< 1%	102	71.3	90	78.9	0.1940
≥ 1%	41	28.7	24	21.1	
Total	143	100	114	100	
CD8					
Score 0–2	14	9.7	8	6.9	0.5049
Score 3–4	131	90.3	108	93.1	
Total	145	100	116	100	
P16					
Score 0–1	3	2.1	3	2.6	1.0000
Score 2–3	142	97.9	114	97.4	
Total	145	100	117	100	

When the cases were scored with the TPS cut-off ≥ 50% overall expression was positive in 81/547 (14.8%) of the cases. In 20/143 (14%) and 14/114 (12.3%) p = 0.5287, of non-HIV and WLWH groups respectively, with no statistically significant differences.

### P16 and CD8

P16 TMA cores were evaluable in 567 samples (8 cores lost). P16 immunostaining was block positive in 546/567 (96.3%), partially staining was observed in 5/567 (0.9%) and in 16/567 (2.8%) cases showed no staining for p16.

CD8 was expressed in 561/563 (99.6%) cases with evaluable cores (12 cores lost). Two cases (0.4%) had no staining for CD8 cell (Table 1), 10/563 (1.8%) had 1–2 CD8 positive stained cells 53/563 (9.4%) had between 3 and 15 CD8 stained cells. In 450/563 (79.9%) more than 15 CD8 cells were positive, 48/563 (8.5%) expressed extreme density of CD8 positive cells (uncountable positive cells), in the TMA core (Fig. 1).

### Ki-67

The proliferative index was assessed, and available 563 samples (12 cores lost). 536/563 (95.2%) expressed Ki-67 in > 50% of cells (score 3), 18/563 (3.2%) expressed Ki-67 in 30–50% of cells (score 2) and 9/563 (1.6%) Ki-67 was expressed in less than 30% (score1).

Regarding HIV status, there were no differences of P16 and CD8 expression between the HIV positive and HIV negative groups (Table 2).

In order to evaluate interactions of the biomarkers and clinical data on PD-L1 expression a multiple regression analysis for each of the PD-L1 CPS/TPS variants was performed. Concerning CPS > 1%, an influence of CD8 expression was found (p = 0.041), concerning TPS ≥ 25% and ≥ 50% no statistically significant influence was found, data not shown.

In multiple regression analyses, Ki-67 proliferation of > 30% did not correlate to any other biomarker expression, nor stage or HIV status.

## Discussion

In the present study we conducted a retrospective analysis of PD-L1 SP263 immunohistochemistry expression in a Mozambican cohort of invasive CC, one of the largest cohorts of SCC from an HIV endemic region. We evaluated the PD-L1 expression based on TPS and CPS for the overall cohort, and further we stratified according to the HIV status of the patients. Additional we also assessed the immunohistochemistry expression of p16, CD8 and Ki-67.

It has been suggested from other studies that HIV infection enhances the carcinogenic risk of other viruses through many underlying mechanisms such as an ineffective immune surveillance and facilitates the proliferation of the oncogenic viruses^36^. The chronic inflammatory state of HIV infection results in upregulation of PD-L1 expression, that leads to CD8 + T cells exhaustion, promoting the evasion of HPV infected cells and allowing them to grow and differentiate in an uncontrolled manner, and to develop dysplasia and cancer^47^. The relationship of HIV-HPV infection in carcinogenesis is well established in the cervix, oropharynx, and anal SCC^2,36,48^.

According to our results, the expression of therapeutical target (PD-L1) is not affected by HIV status of the patients, which implies the potential use of PD-L1 targeted therapy both in women living with or without HIV. The same is valid for the presence of CD8 + T lymphocyte population.

PD-L1 expression found in the present study in CPS (26.3%), was less than reported in other studies in CC, ranging from 32.4 to 80% in cervical SCC^49–55^. Feng et al. that used the TMA approach to perform the PD-L1 immunohistochemistry reported a 32.4% of PD-L1 positive expression^49^. The slightly lower expression of PD-L1 in our study could be related with the heterogenic pattern of expression that have been widely demonstrated in studies using whole slide (WS) preparations, and even though we have sampled three random TMA cores, we could still have missed the spots that were positive in some cases. This indicates that PD-L1 expression still may be systematically underestimated in TMA studies highlighting the fact that TMA is a valuable technique for studies of large cohort but should not be used for the assessment of individual cases.

When comparing the PD-L1 expression in the cases with known HIV status, we found a numerically lower percentage of patients expressing PD-L1 as determined by CPS ≥ 1 (21.1%) and TPS ≥ 25% (16.6%) in the WLWH group compared with the non-HIV women group that expressed little more PD-L1 on CPS ≥ 1 (28.7%) and TPS (20.9%), with non-statistically significant differences of expression between the groups. Previously, differences of PD-L1 expression between WLWH and non-HIV patients have been reported^56^, and PD-L1 expression on the WLWH group was demonstrated on CPS ≥ 1 as 78.6% and TPS ≥ 25% as 31%, thus compared to our results with divergent data concerning especially the CPS, in spite of using identical immunohistochemical assay. A recent study, performed in WLWH and non-HIV patients with *in-situ* and invasive carcinomas, reported lower (16.7%) PD-L1 expression in both groups using SP263 TPS ≥ 50% and 22C3 CPS ≥ 1^57^. Brito et al. found similar results of PD-L1 expression in cervical SCC (around 16% in both WLWH and non-HIV patients) using a threshold of TPS ≥ 50% and CPS > 1)^57^. Our results were even lower when scored in TPS > 50% showing 14% and 12.3% in non-HIV and WLWH groups respectively, and no statistically significant differences were observed.

A number of different factors can explain differences between the studies like methodological differences, characteristics of material used, sampling characteristics, anti retroviral therapy (ART) and HIV burden. It has been hypothesized that in WLWH patients under ART, could express less PD-L1 in the tumour cells as result of the therapy^58^, even though in general it has been stated that WLWH patients are more prone to over-express PD-L1 in the tumour cell as a result of the chronic inflammatory status driven by HIV infection itself, as well as genomic alterations induced by HPV/HIV synergetic effect^2,59^.

In the perspective of immunotherapy, has been suggested from other studies, that WLWH patients with invasive CC could benefit from it, as the side effects are similar to non-HIV patients^32–34^. Furthermore, it was demonstrated in a melanoma study that CD8 + T cells infiltrate density was more predictive of ICI immunotherapy response^60^. The results of our study, showing that in 88.4% of the cases were CD8 + T lymphocytes infiltrate scored 3 (more than 15 cells) and 4 (uncountable positive cells), mainly intra-tumoral infiltrate, raises the possibility that the majority of the patients of the cohort could have had a good response to the treatment with ICI immunotherapy^40,60^, meaning that in the setting of this study, the ICI therapy would be an alternative to improve the survival rates of CC patients.

In the present study we included a large number of CC of both WLWH and non-HIV patients from a country with high prevalence of both HIV, HPV and CC. Since in this study we have used already existing databases as source demographic and clinical data gathering, this implied a series of limitations to this study such as incomplete clinical data, absence of HIV testing status for the whole cohort and absence of quantitative data on the WLWH regarding the viral load, CD4 + cell count, HIV subtype and data on ART (how long in ART and type of drugs used), and absence of FIGO stage for the majority of the patients. Furthermore, even though some methodological challenges concerning immunohistochemistry^45,61^ has been handled, further possible methodological challenges of specimen handling for histopathology^62^ may remain in the present low resource setting.

Even though with the outlined limitations, our data indicates that the documented positive effect of PD-L1 on advanced stage of carcinomas^63^ could be valid also for WLWH patients with SCC in Mozambique. Thus, there is an urgent need for randomized clinical trials, including optimal biomarker analyses, to demonstrate the feasibility and clinical effect of PD-L1 inhibitors in the HIV endemic milieu of sub-Saharan Africa.

## Methods

### Study population, sampling, and samples

Tumour samples as well as demographic, clinical and HIV serology data were obtained from the computer records files of the Pathology Department and from the Hospital-based Cancer Registry databases of the Maputo Central Hospital (MCH), a quaternary and national referral hospital of Mozambique. The HIV serology testing information used in the present study were available in these records. Nationally in Mozambique the HIV testing is performed according to the World Health Organization (WHO) guidelines for high prevalence countries^64^, using rapid diagnostic test (RDT), where the negative result is assigned by one RDT and positive result is followed by second and third different RDTs^65^. The viral load of the patients was not available for any of the cases, as the follow up and treatment for HIV is performed in the primary and secondary level health care facilities.

The Department of Pathology of MCH is responsible for the anatomopathological diagnosis of all specimens coming from the three provinces of the southern region of Mozambique (Inhambane, Gaza and Maputo), and is also a referral for consultation for diagnosis of cases from the whole country. All cases consecutively diagnosed as CC obtained through biopsy or surgical resection, from January 2017 to December 2018 in the Department of Pathology at MCH were retrieved.

All Haematoxylin and Eosin (H&E) stained slides obtained from formalin fixed paraffin embedded (FFPE) tissue blocks were scanned at 40 × brightfield on a Panoramic 250 automatic digital scanner (3D HISTECH Ltd., Budapest, Hungary) at the Department of Clinical Research of Orebro University Hospital (OUH), Sweden, and stored within a storage system from the same supplier at a server facility, fulfilling the general data protection regulation (GDPR). The scanned material and tissue microarray (TMA) slides were assessed and used for evaluation through the Panoramic Case Viewer software version 2.4.0.119028 for Microsoft Windows.

**Figure 2:**
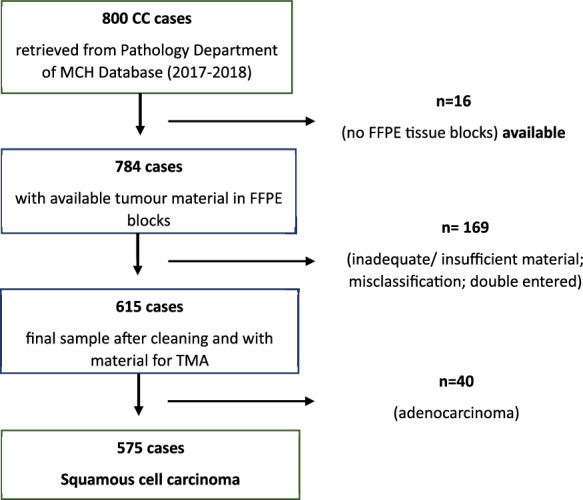
Flowchart of exclusion process within the overall cohort of cervical carcinoma cases.

All cases of invasive CC consecutively diagnosed in the study period, and with sufficient tumour material in FFPE block for extraction of at least three core cylinders of 0.6mm for TMA construction and with no depletion risk of the donor block sample were included to the study. Cases with no available tissue, inadequate or insufficient tumour material in FFPE block for TMA construction, non-invasive carcinoma, wrong coded or double entered were excluded. Out of 615 cases available after review of the blocks, 575 were squamous cell carcinomas, which were our final cohort for this study (Fig. 2).

For TMA construction, the H&E digitalized slides were assessed through the above-described software. Selection of tumour area was performed on those slides by a pathologist (L.L.).

### TMA construction

The areas of interest (AOI) were identified, and the TMA circles markers of 0.6 mm diameter were placed. For each slide, three TMA circle markers were placed. The tissue cylinders punches were performed using the TMA Grand Master 2.8 (3D HISTECH Ltd., Budapest, Hungary), an automated tissue microarrayer. The tissue cylinder cores punched from the donor block were automatically transferred into a new paraffin block called recipient block, according to the manufacturer specifications.

All TMA blocks were sectioned in 4µm thickness and transferred to the glass slide. At least one section per paraffin block was stained with H&E to confirm the histology and to certify the availability of tumour tissue in the slides by a pathologist. Other slides were submitted to immunohistochemistry for PD-L1, CD8, P16 and Ki-67. The sectioning for immunohistochemistry were performed the day before the immunostaining^45,61^.

### Immunohistochemistry performing and scoring

PD-L1 SP263 and CINtec p16 (E6H4) were performed on Benchmark Ultra (Roche diagnostics, Switzerland) according to the manufacturer’s instructions. CD8 (C8/144B) and Ki67 (MIB-1), was performed on DAKO OMNIS (Agilent, Santa Clara, USA), with a polymer-based detection system, according to the manufacturer’s instructions (Agilent, Santa Clara, USA). Positive and negative control tissues were included for all antibodies used in each run. Control tissues were included in each run, tonsil, negative lung SCC and positive lung SCC for PD-L1, tonsil and appendix for CD8 and tonsil for p16 as recommended by the manufacturer. Additional, for PD-L1 SP263, an isotype specific negative control was included. The cases were considered evaluable if at least one TMA core was present.

The PD-L1 expression was scored according to Ventana algorithm for PD-L1 SP263^66^. The tumour proportion score (TPS) is calculated by counting PD-L1 stained tumour cells divided by the total number of tumour cells, PD-L1 was considered positive when tumour cells had partial or complete circumferential brown staining on the cellular membrane at any intensity. The TPS was further categorized into two groups: high/positive when ≥ 25% of tumour cells exhibit membrane staining; and low/negative when < 25% are stained^56,66,67^, a ≥ 50% cutoff was also used^68^ and the combined positive score (CPS) was also assessed using the cutoff of ≥ 1. The CPS was defined by sum of the tumour cells and mononuclear immune cells stained with PD-L1 divided by the total number of tumour cells, then multiplied by 100^39^.

P16, was scored as positive when both cytoplasmatic and nuclear brown staining was present. According to the extent of positivity, p16 was scored on a four-grade scale as: strong diffuse (3 +), moderate intensity (2 +) and weak sporadic or mild intensity (1 +) or no visible staining (0). Depending on the percentage of positive cells, staining was graded in a semi-quantitative manner, as: Score 0: negative cells; Score 1 – less than 10% of positive cells; Score 2–10 to 50%); Score 3– > 50%^69^.

The scoring of CD8 was semi-quantitative; CD8 was considered as positive when there was brown staining of the membrane and cytoplasm of lymphocytes, and was scored in five grade scale: score 0: no CD8 + cell staining; score 1: (low density): 1–2 CD8 + stained cells per core); score2 (moderate density): 3–15 CD8 + stained cells per core; Score3 (high density): more than 15 countable CD8 + stained cells per core); or score 4 (extreme density): uncountable CD8 + stained cells per core^21^.

Ki67 was scored as positive when brown staining was present in the nucleus at any intensity. The positive staining was further categorized in three-grade scale in a semi-quantitative manner as: Score 1: 10–30% stained cells (Low proliferation); Score2: 30–50% stained cells (moderate proliferation); Score3: > 50% (high proliferation)^70^.

For all challenging cases, scoring was discussed, and consensus was reached.

### Statistical analysis

Descriptive statistics and assessment of differences between groups, the later using Fishers exact test as well as Multivariate Logistic Regression Analyses with statistical significance considered to be significant with p < 0.05. SPSS (IBM SPSS v 28.0.0.0).

### Ethical considerations

All the experimental protocols were approved by the National Bioethics Committee for Health (CNBS) board of Mozambique under registration number 114/CNBS/2019 and Swedish Ethical Review Authority under registration number 2023-01674-01-423571, according to the Helsinki Declaration in human subject research. In Mozambique, the Ethical board (joint committee of the Faculty of Medicine, Eduardo Mondlane University and Maputo Central Hospital) at the university reinforced the project, but since the projects included the use of human biological material an approval from the governmental authority, National bioethical committee, was requested. In Sweden, ethical approvals are granted by a governmental authority which is separate and independent from the universities and other research bodies and health care providers. Thus, Örebro university does not have any separate ethical board. The tissue material consists of formalin fixed paraffin embedded (FFPE) blocks of invasive cervical cancer samples from biopsy or surgery obtained and sent to the Pathology Department of Maputo Central Hospital for diagnostic purposes on the years 2017 and 2018. The informed written consent requirement was waived by the National Bioethics Committee for Health (CNBS) board of Mozambique for the study protocol. Patient consent was waived due to the reason of being retrospective study in which the samples used are cervical cancer paraffin blocks stored after diagnosis in Department of Pathology of Maputo Central Hospital. The cases represent patients from many parts of Maputo City, southern region, or other regions of Mozambique, some of whom deceased and without any information regarding address or telephone numbers on the histopathology request form, that makes impractical to obtain direct informed and likely impossible. The tissue samples were stored for future medical studies, and furthermore strictly anonymized before inclusion in the present study. No further sampling, treatment or contact with the patients was needed. The new information received would not change any potential forthcoming treatment or outcome for the individual patients.

## Data Availability

All data generated or analysed during this study are included in this article. Further enquiries can be redirected to the corresponding author.
